# How to improve smoking cessation support for pregnant women? Guideline implementation in The Netherlands

**DOI:** 10.1093/heapro/daaf084

**Published:** 2025-06-18

**Authors:** Stella Weiland, Danielle E M C Jansen, Gera A Welker, Marjolein Y Berger, Jan Jaap H M Erwich, Lilian L Peters

**Affiliations:** Department of Primary and Long-Term Care, University Medical Center Groningen, University of Groningen, Hanzeplein 1, 9713 GZ Groningen, The Netherlands; Midwifery Academy Amsterdam Groningen, InHolland, Dirk Huizingastraat 3-5, 9713 GL Groningen, The Netherlands; Midwifery Science, Amsterdam UMC Location Vrije Universiteit Amsterdam, Van der Boechorstraat 7, 1081 BT Amsterdam, The Netherlands; Department of Primary and Long-Term Care, University Medical Center Groningen, University of Groningen, Hanzeplein 1, 9713 GZ Groningen, The Netherlands; Department of Sociology and Interuniversity Center for Social Science Theory and Methodology (ICS), University of Groningen, Grote Kruisstraat 2/1, 9712 TS Groningen, The Netherlands; University Medical Center Groningen, UMC-Staff Policy and Management Support, University of Groningen, Hanzeplein 1, 9713 GZ Groningen, The Netherlands; Department of Primary and Long-Term Care, University Medical Center Groningen, University of Groningen, Hanzeplein 1, 9713 GZ Groningen, The Netherlands; Department of Obstetrics & Gynecology, University of Groningen, University Medical Center Groningen, Hanzeplein 1, 9713 GZ Groningen, The Netherlands; Department of Primary and Long-Term Care, University Medical Center Groningen, University of Groningen, Hanzeplein 1, 9713 GZ Groningen, The Netherlands; Midwifery Academy Amsterdam Groningen, InHolland, Dirk Huizingastraat 3-5, 9713 GL Groningen, The Netherlands; Midwifery Science, Amsterdam UMC Location Vrije Universiteit Amsterdam, Van der Boechorstraat 7, 1081 BT Amsterdam, The Netherlands

**Keywords:** implementation study, smoking cessation, participatory action research, mixed-methods

## Abstract

This study aimed to develop and evaluate plans for the implementation of the Dutch guideline ‘Treatment of tobacco addiction and smoking cessation support for pregnant women’. Participatory action research was used for the development and evaluation of implementation plans for maternity collaboration units in the north of the Netherlands. Mixed methods were used to evaluate the implementation by using the Reach, Effectiveness, Adoption, Implementation and Maintenance framework. The maternity collaboration units implemented the intervention to refer pregnant women who smoke to a counsellor from addiction care. Twenty-one of the 50 midwifery care practices (42%) and two of the five obstetrics departments (40%) referred women to addiction care. The results showed that of the 558 women who smoked during pregnancy in 2021, 73 women (13%) were referred to addiction care, 58 (10%) started a coaching trajectory and 12 women of the 48 (25%) who finished a coaching trajectory stopped smoking. The results of interviews and focus groups gave insight into the challenges for referral and indicated that the communication between the midwife/counsellor and the pregnant woman is important for counselling. A minority of maternal care professionals referred women to a counsellor from addiction care and a small percentage of women managed to stop smoking. Opportunities in the repetition of implementation strategies and increasing skills in motivational interviewing for maternity care professionals could improve adoption of interventions in future implementation. To increase the effectiveness of the intervention, the counsellors could consider combining their counselling with nicotine replacement therapy, feedback or incentives.

Contribution to Health Promotion

- In this study a smoking cessation support intervention is implemented for pregnant women with the aim to contribute to decreasing the prevalence of women who smoke during pregnancy.
- Some pregnant women were successful in smoking cessation with the support of a counsellor from addiction care.
- The results indicate that the repetition of implementation strategies and increasing skills in motivational interviewing could improve future adoption of the intervention.

## INTRODUCTION

In the Netherlands, 8% of women smoke at some point during pregnancy (Scheffers-van Schayck *et al*. 2021). Smoking during pregnancy is an important preventable risk factor for complications; women who stop smoking during pregnancy decrease their risk for adverse outcomes such as miscarriage and stillbirth (Abraham *et al*. 2017). Smoking cessation support can increase the proportion of women who quit smoking during pregnancy (Lumley *et al*. 2009; Chamberlain *et al*. 2017).

**Figure 1.**
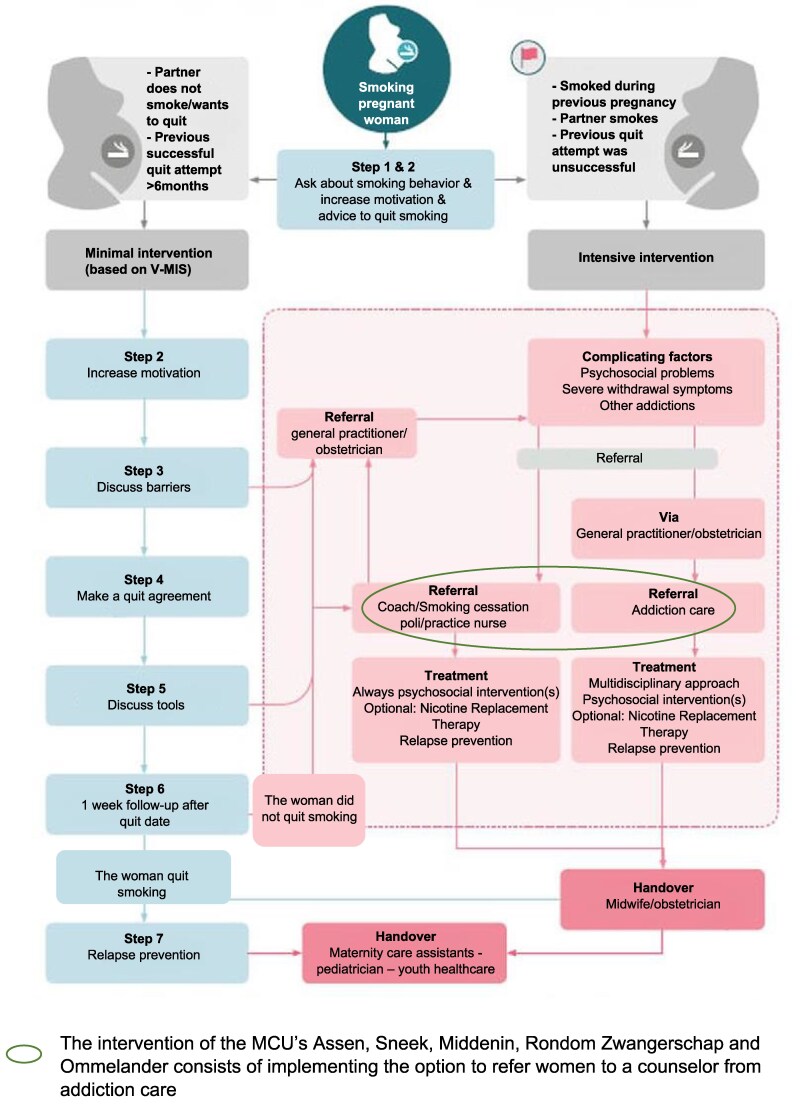
Graphical representation of the guideline including the focus of the intervention, adapted and translated to English, from the Trimbos-Institute.

The Dutch guideline ‘Treatment of tobacco addiction and smoking cessation support for pregnant women’ from the Trimbos-Institute includes specific recommendations for maternity care professionals (MCP’s, e.g. midwives and obstetricians) on how to support pregnant women with smoking cessation (Fig. 1) (Trimbos-instituut 2017).

One of the main recommendations of the guideline is to provide behavioural counselling via the Minimal Intervention Strategy for Midwives (V-MIS). The V-MIS consists of seven steps to support pregnant women and their partners with smoking cessation, ranging from discussing smoking behaviour to increasing motivation for relapse prevention (Trimbos-instituut 2017). Women can also be referred to a trained nurse practitioner working in general practice or to a smoking cessation counsellor, such as a counsellor from addiction care, for behavioural counselling (Trimbos-instituut 2017). The guideline also describes the use of nicotine replacement therapy, e-health, and the electronic-cigarette (Trimbos-instituut 2017).

Two studies from 2019 investigated the degree of implementation of this guideline in the north of the Netherlands (Meije and van Aerde 2019; Warmelink *et al*. 2021). The results of these studies indicated that 21%–45% of the intended users (MCP’s) of the guideline were not familiar with the guideline, 33%–37% were familiar with the guideline but did not use it, and only 22%–31% used the guideline (Meije and van Aerde 2019; Warmelink *et al*. 2021). A few studies evaluated specifically the implementation of the V-MIS and reported a poor implementation in daily midwifery care (Oude Wesselink *et al*. 2015; Hopman *et al*. 2019). Other parts of the guideline, such as referral to a trained nurse practitioner and a smoking cessation counsellor, have not been evaluated. Until now, smoking cessation counselling provided by a counsellor from addiction care was not implemented in the north of the Netherlands.

Because of the effectiveness of smoking cessation support, optimal implementation of the guideline in daily practice is essential (Lumley *et al*. 2009; Chamberlain *et al*. 2017). Improved adherence to the guideline can advance the provision of smoking cessation support by MCP’s and ultimately decrease smoking during pregnancy. From implementation research, we know that implementation success is influenced by tailoring the implementation to the barriers and facilitators of the target groups (Powell *et al*. 2012). Therefore, we hypothesized that plans for implementing the guideline were needed in the Maternity Collaboration Units (MCU) located in the three northern Dutch provinces of Groningen, Friesland and Drenthe. In an MCU, MCPs collaborate in a region, often centred around a hospital. We focused on the north of the Netherlands because relatively many pregnant women smoke in this region, compared to other parts of the Netherlands (Volksgezondheidenzorg.info 2017; Scheffers-van Schayck *et al*. 2019). This is related to the relatively low socioeconomic status of residents in the north (Volksgezondheidenzorg.info 2017; Scheffers-van Schayck *et al*. 2019).

The aim of this study was to develop and evaluate tailored implementation plans for the guideline on smoking cessation support for pregnant women in seven MCU’s in the north of the Netherlands. These plans were designed based on the specific barriers, facilitators, and needs identified within each MCU. By evaluating the implementation of these tailored plans, we aim to understand what works and what does not, thereby gaining insights into how tailored implementation strategies can enhance the uptake of a clinical guideline. The implementation was evaluated by using the Reach, Effectiveness, Adoption, Implementation and Maintenance (RE-AIM) framework (Glasgow *et al*. 1999).

## MATERIALS AND METHODS

### Setting and study population

In this study we focus on seven MCU’s in the northern provinces of Groningen (MCU Martini, MCU Ommelander), Friesland (MCU Sneek, MCU Rondom Zwangerschap, MCU Middenin), and Drenthe (MCU Assen and MCU Stadskanaal-Hoogeveen-Emmen). Two MCP’s (midwives and/or obstetricians) per MCU, who were in charge of the smoking cessation policy within the respective MCU, were recruited to participate in the study. By granting consent to participate in the study, they acted as representatives for their MCU and were the point of contact for the researchers and the other MCP’s of the MCU. The representatives received an annual fee of €250 to participate in the study.

All women who smoked during pregnancy were eligible to participate and were recruited by MCP’s of the MCU’s. Pregnant women were informed about the study in person by the MCP’s and via an information letter. Women signed an informed consent form for their participation in the study.

### Study design

This study used participatory action research (PAR) as a research design. PAR is a research methodology in which researchers and stakeholders closely collaborate to achieve change (Baum *et al*. 2006). PAR increases the likelihood of the success of implementation of an intervention (Wallerstein and Duran 2010; Wensing and Grol 2019). To tailor the implementation strategies to the specific contexts of each MCU, the barriers, facilitators and needs were discussed for each MCU and implementation plans were developed in collaboration with several stakeholders: two midwives and/or obstetricians per MCU, physicians and counsellors from addiction care, an implementation expert, pregnant women (who smoked at some point before or during pregnancy and participated in interviews), and two experts by experience in poverty and social exclusion (Damschroder and Hagedorn 2011).

Our first step in developing and evaluating plans for smoking cessation support involved conducting interviews to gain insight into women’s and their partners’ experiences and needs with smoking cessation support in the north of the Netherlands. The methods and results of the interviews were described in a previous paper (Weiland *et al*. 2021). Additionally, we conducted three focus groups with MCP’s in the provinces of Groningen (*n* = 7), Friesland (*n* = 7) and Drenthe (*n* = 6) to investigate their experienced barriers, facilitators, and needs with providing smoking cessation support according to the guideline (Edens 2020). The focus groups were moderated by an independent researcher from ‘Zorgbelang’ Groningen, an organization, which represents the voices of people who make use of healthcare.

The second step was discussing for each MCU which form of cessation support (intervention) would fit best for the barriers and facilitators and needs of each MCU. After the intervention per MCU was determined, tailored implementation strategies were chosen per MCU based on the barriers, facilitators, and needs that were found during the interviews and focus groups (Damschroder and Hagedorn 2011; Powell *et al*. 2012). Then, the intervention and implementation strategies were written down in an implementation plan per MCU to guide the implementation for each MCU. Execution of the implementation plan took place in 2020 and 2021.

The third step was evaluating the actual implementation using the Reach, Effectiveness, Adoption, Implementation, and Maintenance (RE-AIM) framework (Glasgow *et al*. 1999). The SQUIRE 2.0 checklist was used to report this study (Supplementary File S1) (Ogrinc *et al*. 2016).

### Data collection for the evaluation of the implementation

We used quantitative and qualitative data to evaluate the implementation according to the RE-AIM framework (Table 1) (Glasgow *et al*. 1999). We used mixed methods because they provide the opportunity to understand the results of the implementation in a specific context (Albright *et al*. 2013). In this study we did not take maintenance into account because data on long-term outcomes were not available during the study period. All data collection was done during or after the implementation.

**Table 1.** Outcome measures and methods for the evaluation of the implementation based on the RE-AIM framework (Glasgow *et al*. 1999).

RE-AIM framework (Glasgow *et al*. 1999)	Operationalization outcome measures	Method
Reach ‘The number of people who are willing to participate in an intervention’	1. Total women who received maternal care 2. Total women who smoked during pregnancy 3. Total women who participated during the implementation in 2021	• The registry data of electronic patient files of 2021 provided by midwifery care practices and departments of obstetrics of hospitals that participated in the implemenation • Data from the logbooks of addiction care counsellors
Effectiveness ‘The impact of an intervention’	The impact of the implementation on the smoking behaviour of pregnant women: continued smoking, reduced number of cigarettes or stopped smoking during pregnancy	Data from the logbooks of addiction care counsellors and the researcher (SW)
Adoption ‘The number of people who are willing to initiate an intervention’	The number of midwifery care practices and departments obstetrics in the hospital of each MCU that participated in the implementation	Data from the logbooks of addiction care counsellors and the researcher (SW)
Implementation ‘People’s fidelity to the elements of an intervention and adaptations to the intervention and strategies’	1. The execution of the implementation strategies as intended 2. The implementation degree of the guideline among MCP’s 3. Implementation of the guideline as perceived by pregnant women 4. Experiences of pregnant women and MCP’s during the implementation	• Two online questionnaires targeted at pregnant women who smoked and maternal care professionals who provide smoking cessation support • Data from the logbooks of addiction care counsellors • Results of the interviews with maternal care professionals that participated in the implementation and women who received counselling by the counsellors of addiction care • Results of the focus groups with maternal care professionals that participated in the implementation

First, with registry data of electronic patient files from MCP’s we aimed to gain insight into the reach of the implementation, the prevalence of women who smoked during pregnancy. The data consisted of numbers of the total women receiving care and the number of women who smoked during pregnancy in 2021. These data were supplied by primary midwifery care practices and departments of obstetrics and gynaecology of hospitals of the MCU’s that participated in the implementation.

Second, to evaluate the implementation of the guideline among MCP’s we used the data from the online survey ‘Monitor Smokefree start’ initiated by the Trimbos-Institute in 2021. We analysed the item ‘Are you familiar with the Trimbos-Institute guideline?’ with the answer options ‘no’, ‘yes but I don’t work with it (yet)’, and ‘yes and I work with it’ (Meije and van Aerde 2019). The online survey was distributed by the researchers and the Trimbos-Institute among the MCP’s of the northern MCU’s in January 2021. The MCP’s that were in charge of the smoking policy of an MCU were invited to participate in the survey and were asked to distribute the survey among their colleagues.

Third, we investigated the implementation of the guideline as received by pregnant women who smoke with another online survey. We opted for a survey because we aimed to reach as many pregnant women who smoke as possible. We recruited women who had been pregnant and smoked before or during pregnancy in the period from 2019 until 2022 via Facebook pages targeted at pregnant women and mothers in the north of the Netherlands. The survey was pilot tested among four pregnant women, two experts by experience in poverty and social exclusion and two students from the midwifery academy Groningen. Based on the pilot, some questions were re-formulated to enhance clarity of the questions. The online survey focused on women who smoked during pregnancy and consisted of items about women’s smoking behaviour, the information they received about smoking cessation care from the midwife and/or obstetrician and if they made use of smoking cessation support.

Fourth, to investigate the reach, adoption, implementation, and effectiveness of the implementation, the MCP’s involved in the implementation (midwives, obstetricians, and counsellors) and the researcher (SW) kept logbooks. The logbooks contained information about the number of women who participated in the intervention, the effect of the intervention on women’s smoking behaviour, the adoption of the intervention by the MCU’s and the execution of the implementation strategies as planned.

The qualitative data collection was aimed at gaining insight into the experiences of included women and MCP’s during the implementation. Semi-structured interviews were conducted with women who smoked during pregnancy (*n* = 21). The interviews were performed from March 2020 until March 2022 and lasted on average 22 min. Semi-structured interviews were also conducted with MCP’s (*n* = 9) and were focused on their experiences during the implementation. These interviews were performed in the period from March 2020 till January 2022, the interviews lasted between 14 and 64 min.

In addition, three focus groups were performed with the MCP’s per MCU that were in charge of the smoking cessation policy within the respective MCU in Groningen (*n* = 5), Friesland (*n* = 6), and Drenthe (*n* = 5) in November 2021. The aim of the focus groups was to evaluate their experiences during the implementation. An independent researcher from the foundation ‘Zorgbelang’ Groningen moderated the focus groups. The focus groups each lasted 90 min.

### Analysis

Descriptive statistics (numbers and proportions) were used to report all quantitative outcomes according to the RE-AIM Framework (Glasgow *et al*. 1999) in SPSS version 26.0 (SPSS Inc., Chicago, IL, USA).

The interviews and focus groups were audio recorded with permission of the participants and transcribed verbatim. For both the interviews and focus groups, we conducted a (reflexive) thematic analysis following the six-phase approach by Braun and Clark (Braun and Clarke 2006, 2024), grounded in a constructionist epistemology. This approach views meaning and experience as socially produced and acknowledges the active role of the researcher in the analytic process. First, two researchers (SW and WB) read all interview and focus group transcripts multiple times to familiarize with the data. Subsequently, SW and WB independently coded each transcript and generated initial inductive codes in Atlas.ti 8.4. Then, SW and WB discussed their coding decisions with the aim to identify potential themes in the data. Some codes were clustered and other codes were split to form categories, themes, and sub-themes.

## RESULTS

### First step: experienced barriers, facilitators, and the needs of MCP’s regarding smoking cessation support

The independent researcher from ‘Zorgbelang’ Groningen wrote a report about the results of the focus groups (Edens 2020). The results indicated that MCP’s experience a lack of referral options and a lack of knowledge about referral options, a lack of skills in motivational interviewing, a lack of time for midwives during their consultation to provide smoking cessation support, and a lack of collaboration in the smoking cessation support of pregnant women as main barriers. Having a maternal care professional who was responsible for the smoking cessation policy within the respective MCU was identified as the main facilitator (Edens 2020). The results of the focus groups indicated that MCP’s from the seven MCU’s needed clarity about who is responsible for smoking cessation support, a referral option with expertise in providing smoking cessation support, a clear referral process, and collaboration between MCP’s in the provision of support (Edens 2020).

### Second step: selection of intervention and implementation strategies

The smoking cessation intervention that was chosen by the seven MCU’s Assen, Sneek, Stadskanaal-Hoogeveen-Emmen, Martini, Middenin, Rondom Zwangerschap and Ommelander was the option to refer women to a counsellor from addiction care for behavioural counselling (not including the prescription of nicotine replacement therapy), which is part of the guideline (Fig. 1) (Trimbos-instituut 2017).

This intervention was tailored to the needs addressed by the seven MCU’s for a clear referral option to someone who is responsible for smoking cessation support and who has expertise in support provision. This particular intervention, described in the guideline, was not implemented yet. Multiple implementation strategies (Table 2) were used to enhance the implementation, tailored to the barriers, facilitators and needs of each MCU. Strategies that focused on engaging the MCU’s in the implementation of the intervention and increasing MCP’s knowledge about the intervention and the procedure for referral were similar for all seven MCU’s. First, the midwives and obstetricians of all seven MCU’s were informed about the implementation of the referral option in person, via e-mail and/or by telephone. Second, MCP’s from all seven MCU’s received an information leaflet about the procedure to be followed when referring to counsellors from addiction care. This procedure meant that MCP’s, with the woman’s approval, sent the woman’s e-mail address and phone number to the counsellor from addiction care, who subsequently reached out to the woman. The counsellor contacted the woman and together they made a tailored smoking cessation plan, which involved setting a goal (i.e. a stop date, reducing the number of cigarettes) and determining the mode and frequency of contact (telephone, WhatsApp, or in person).

**Table 2.** Implementation strategies used for the implementation of referral to a counsellor from addiction care.

MCU’s	Barriers, facilitators and needs	Implementation strategies
All seven MCU’s	Having a MCP who is in charge of the smoking cessation policy for the MCU	• Having two MCP’s per MCU who acted as representatives for the MCU and were paid an annual fee for their participation in the development and implementation of the implementation plan
	A lack of knowledge about who is responsible for providing smoking cessation support A lack of time during the consultation to provide smoking cessation support	• Providing information about referral to a counsellor of addiction care during an MCU meeting in person • Personal introduction of the counsellor of addiction care in an MCU meeting • Distributing a flyer with information about the counsellor of addiction care via e-mail
	A lack of knowledge about how to refer women	• Providing information about the referral process to a counsellor of addiction care via e-mail and by telephone • Distributing a flyer with example sentences to use for referral to a counsellor of addiction care • Interim visits of the researchers to an MCU meeting to provide information about the progress of referrals to the counsellor
MCU Assen	A lack of skills in motivational interviewing	• Providing the e-learnings ‘Smokefree Start’ and ‘Comprehensible Communication’ and the on-site training ‘Smokefree Start’ to maternal care professionals
MCU Rondom Zwangerschap	A lack of knowledge about referral options as described in the guideline	• Developing a flowchart with referral options and distributing the printed flowchart with referral options among maternal care professionals

Third, some MCU’s required additional strategies, specifically tailored to the MCU’s barriers and facilitators. The MCU Rondom Zwangerschap received a flowchart with all referral options as described in the guideline ‘Treatment of tobacco addiction and smoking cessation support for pregnant women’ from the Trimbos-Institute (Trimbos-instituut 2017). This implementation strategy addressed their experienced barrier about a lack of knowledge of referral options. MCP’s of the MCU Assen experienced a lack of skills in motivational interviewing as a barrier to referring women for smoking cessation support. Therefore, they received training in motivational interviewing by following two e-learnings ‘Smokefree Start’ and ‘Comprehensible Communication’, and subsequently the on-site training ‘Smokefree Start’ from the Trimbos-Institute.

### Third step: evaluation of the implementation using the RE-AIM framework

#### Reach

Of the 8890 pregnant women who received care in the seven MCU’s in 2021, 558 women (6.3%) smoked during pregnancy and 343 women (3.9%) stopped smoking prior to pregnancy or when discovering their pregnancy. Seventy three of the 558 pregnant women who smoked (13.1%) were referred by the midwife or gynaecologist for smoking cessation support from a counsellor from addiction care in 2021. Of these women, 58 (79.5%) women started a coaching trajectory, and 15 (20.5%) did not reply to the contact attempts of the counsellors or indicated that they preferred to stop smoking without expert support. From the 58 women who started a coaching trajectory, 48 women finished the coaching trajectory (82.8%).

#### Effectiveness

Of the 48 women who finished the coaching trajectory, 12 women (25%) stopped smoking during pregnancy, 21 women (43.8%) reduced the number of cigarettes smoked, and 3 women (6.3%) did not change their smoking behaviour. The remaining 12 women (25%) were lost to follow up.

#### Adoption

In total 66 midwifery care practices and seven obstetrics departments collaborate in seven MCU’s in the north of the Netherlands. Of those, 50 midwifery care practices (75.8%) and five obstetrics departments (71.4%) indicated willingness to refer pregnant women who smoke to a counsellor from addiction care. In total 21 of the willing 50 midwifery care practices (42%) and two of the five willing obstetrics departments (40%) actually referred women to a counsellor from addiction care (Table 3). From the MCU Assen, nine midwives and obstetricians followed the training ‘Smokefree Start’.

**Table 3.** Overview the adoption of the implementation to refer women to a counsellor of addiction care.

Adoption	Total	Assen	SHE	Sneek	Middenin	Rondom Zwangerschap	Martini	Ommelander
Number of midwifery care practices of a MCU^a^	66	11	10	10	17	14	9	6
Number of midwifery care practices that indicated to be willing to refer women to a counsellor of addiction care^a^	50	9	7	6	10	10	8	5
Number of midwifery care practices that actually referred women to a counsellor of addiction care^a^	21	2	6	2	4	1	5	4
Number of departments obstetrics hospital of a MCU	7	1	1	1	1	1	1	1
Number of departments obstetrics hospital that were willing to refer women to a counsellor of addiction care	5	1	1	1	1	0	1	0
Number of departments obstetrics hospital that actually referred women to a counsellor of addiction care	2	1	0	0	0	0	1	0

^a^The numbers of the MCU’s do not add up to the total because some midwifery care practices are part of several MCU’s.

#### Implementation

*The execution of the implementation strategies as intended*: Not all midwifery care practices that initially committed to the implementation managed to collect the registry data that was necessary to evaluate the reach of the implementation. The main reason for this was the heavy burden on midwifery care practices during the Covid-19 pandemic. Another reason was that they were occupied with other (research) projects, such as the Very Brief Advice, that also has a focus on reducing smoking (Papadakis *et al*. 2020). As a result, 15 primary midwifery care practices (30%) and three obstetric departments of hospitals (60%) did not collect the registry data.

During the implementation process we made some adaptations to the implementation strategies based on interviews with MCP’s and counsellors from addiction care. First, the counsellors indicated that some women did not answer their telephone. Therefore, in the information letter to pregnant women we added that the counsellor would call with an anonymous number. The counsellors observed that this adaptation led to an increase in women who picked up their phone. However, it did not result in an increase of women who started a coaching trajectory. Second, because of the increasing number of referrals, more counsellors were recruited to support pregnant women with smoking cessation. Third, because more counsellors were recruited, there was a need from the counsellors from addiction care to centrally manage referrals. Therefore, a special e-mail address was created for all referrals of women to addiction care.

The MCP’s also indicated that they would like to have more regular contact with the counsellors to share their experiences with the smoking cessation process of women. Therefore, the counsellors used a feedback form (including information about frequency of contact and the smoking behaviour of women) which was sent to the midwives and obstetricians after finishing the coaching trajectory, with consent of the women.

Furthermore, some implementation strategies where repeated a few times for MCP’s of the hospital of the MCU Martini, because the representative of the MCU noticed that colleagues in the organization forgot about the intervention. Therefore, the researcher (SW) attended another MCU meeting to provide information about the intervention and the information was provided again by e-mail by the representative of the MCU. In the weeks after repeating this strategy, the number of referrals from the hospital slightly increased.

We did not observe differences in the number of referrals from the MCU’s that had additional implementation strategies, namely a flowchart with referral options and additional training in motivational interviewing, compared to the other MCU’s.

*Implementation degree of the guideline among MCP’s*: In total, 63 MCP’s from the seven involved MCU’s responded to the online survey ‘Monitor Smokefree Start’ initiated by the Trimbos-Institute in 2021 (Willemse *et al*. 2021). Twenty-two respondents (34.9%) indicated that they knew the guideline and used it in daily practice, 26 respondents (41.3%) did not know the guideline, and 15 respondents (23.8%) answered ‘yes, but I don’t use it (yet)’.

*Implementation of the guideline as received by pregnant women*: There were 86 respondents of the online survey, spread across the provinces of Groningen, Friesland, and Drenthe, who indicated they had smoked in the three months before their pregnancy. Of the 86 women, 51 stopped smoking (59.3%) and 35 (40.7%) continued smoking during their pregnancy. Of the 35 women who smoked, 24 (68.6%) were informed about smoking cessation support options in the period from 2019 till 2022. Of these 24 women, 10 (41.7%) indicated that they actually made use of (combinations of) smoking cessation support: a counsellor from addiction care (*n* = 3), telephone-based counselling ‘Smokefree Parents’ (*n* = 1), a trained nurse practitioner working in general practice (*n* = 4), smoking cessation support apps (*n* = 3), nicotine replacement therapy (*n* = 2) or other options (*n* = 1).

*Experiences of pregnant women and MCP’s during the implementation*

Themes: From the analysis of the interviews and focus groups we derived the following two themes: (i) Referral can be a challenge and (ii) Communication between midwife/counsellor and pregnant woman seems crucial for smoking cessation counselling (see Table 4 for the code tree).

**Table 4.** The code tree of the interviews and focus groups with MCP’s and with women.

Theme	Sub-theme	Quote
1. Referral can be a challenge	Organization of care	‘Well my expectation was perhaps that we could include a little more people, that it would live a little more on our ward’ (obstetrician)
	Lack of motivation	‘In our area the motivation to quit is also very low. And I think that’s related to the society they live in, the neighborhood they live in, everyone around them smokes. Especially partners, who are then even less motivated’. (midwife)
	Addiction care	‘The pregnant women I spoke to, ehm were frightened by (addiction care institution) in particular, ehm it sounds pretty intense, I think, for pregnant women and that ehm they are also quickly afraid that they will get a label’. (midwife)
2. Communication between midwife/counsellor and pregnant woman seems crucial for smoking cessation counselling	Pressure	‘Well sometimes people don't respond to their phones. They don't answer the phone. Sometimes we don't have an email address so you just can't email or they don't respond to the emails. […] But then in practice it usually turns out that those pregnant women already have doubts and eh- Actually, they just don't want smoking cessation counselling’. (counsellor addiction care)
	Contact in person	‘I think we had contact in real life two or three times, and after that it was by phone and at some point we also texted. […] Well, of course it was more of an incentive for me to go there. So I found that more pleasant myself, but yes, with the corona that was no option’. (pregnant women)
	No judgement	‘I think that’s very important that just uhm people feel understood because the whole world is already against you if you smoke during pregnancy’. (pregnant woman)

## THEME 1. REFERRAL CAN BE A CHALLENGE

Clinical midwives and obstetricians expressed that, for them, the execution of the implementation strategies seems to be more challenging than for primary care midwives. They expressed that this might be related to the organization of care in the hospital, with relatively many midwives and obstetricians. Therefore, an obstetrician indicated the need to repeat the implementation strategies every now and then.

That makes it quite difficult indeed, (other obstetrician) also had difficulty with that and gave a nice presentation a few times at a given moment and emphasized again how important it is and how easy it actually is to refer, that it is safe to do. And then we had a few more referrals. (obstetrician)

The midwives and obstetricians expressed that referral is challenging in case of women who are not motivated to stop smoking. They indicated that, some women do not want to discuss smoking cessation and will continue smoking. Another challenge for referral is the stigma surrounding addiction care. Midwives mentioned that the opinion that only people with severe addictions go to addiction care, influenced women’s decision not to make use of this smoking cessation support.

We notice that ehm that for many pregnant women, the name (addiction care institution) evokes negative associations. If you explain that, there is a lot of expertise at (addiction care institution), they will understand that, but the threshold is still too high to go along with it. (midwife)

## THEME 2. COMMUNICATION BETWEEN MIDWIFE/COUNSELLOR AND PREGNANT WOMAN SEEMS CRUCIAL FOR SMOKING CESSATION COUNSELLING

Women indicated in the interviews that the communication with the midwife influenced their decision to make use of counselling. Some women who agreed to be referred to a counsellor from addiction care expressed that the information about counselling from the midwife and/or obstetrician was not sufficiently provided. Women also expressed that they sometimes experienced pressure from the midwife or obstetrician to be referred to addiction care.

But she kept bringing it up again and then at a certain moment I said oh okay, […] let (name counsellor) call or text me and then ehm, I'll see ehm what I will do (woman who smoked during pregnancy)

The contact with the counsellor seems to matter for the success of counselling. The counsellors from addiction care expressed that the Covid-19 pandemic influenced their experiences with coaching pregnant women with smoking cessation. Due to the pandemic all counselling was performed via telephone or WhatsApp instead of face-to-face consultations, which might have caused women to end the coaching trajectory prematurely. Counsellors indicated that women were less likely to stop the coaching trajectory prematurely when at least the first contact was in person.

When that contact is good, you can simply start counselling much better. Also by phone afterwards. Because then you know what you look like and uh yes then you also know when someone is joking or not, because then you just know each other (…). And when it’s only with video calling, it’s just less. (counsellor addiction care)

Almost all women who received expert smoking cessation support from a counsellor from addiction care indicated that they liked the way the counsellor approached them, in a very open and understanding way without judgement. Women felt comfortable to discuss their smoking behaviour and their struggles and felt that the counsellor supported them with smoking cessation.

That it’s just really fitted to my needs. I could indicate eh how and why and that she could respond with: ‘Well, hey, maybe you can try this or this’. It wasn't like, ‘you have to do this and you don't have to’ that, so I liked that. (pregnant woman)

## DISCUSSION

In this study we developed and evaluated plans for the implementation of the guideline regarding the smoking cessation support of pregnant women in the north of the Netherlands. Seven MCU’s chose to implement the intervention to refer pregnant women who smoke to a counsellor from addiction care.

There are many possible explanations as to why a minority (40%) of the MCP’s actually referred women and a minority of women (13%) made use of counselling from addiction care. A first explanation could be that not all MCP’s discuss smoking with pregnant women. This was also found in another Dutch study which reported that 40% of pregnant women have discussed their smoking behaviour with a MCP (Scheffers-van Schayck *et al*. 2021). A reason why not all MCP’s discuss the smoking behaviour of women could be that they experience a lack of knowledge and skills in motivational interviewing (Derksen *et al*. 2019). Therefore, there could be an opportunity in increasing the adoption of the intervention by improving skills in motivational interviewing. Although we did not observe differences in referral rate for the MCU that received a training in motivational interviewing, we also do not know if all MCP’s discussed smoking with women throughout pregnancy. Furthermore, other factors that were not anticipated, such as fewer contact moments due to the Covid-19 pandemic or a lack of time (Hopman *et al*. 2019; Scheffers-van Schayck *et al*. 2021), might have influenced referrals.

The implementation of an intervention is complex. Although PAR was used to tailor the implementation strategies to the barriers, facilitators and needs of the MCU’s, this did not lead to large differences in results per MCU. A reason for this could be that the pre-intervention context of the MCU’s (e.g. already trained in motivational interviewing or presence of a smoking cessation policy) influenced the implementation. We did observe an increase in referrals after repeating some strategies for the MCU Martini. This result emphasizes the importance of the repetition of strategies. Additionally, minor adjustments to the implementation plans—such as establishing a dedicated e-mail address for managing referrals and informing women that counsellors would call from an anonymous number—proved beneficial to the implementation process.

In the interviews, the MCP’s provided another explanation why a minority of women were referred to a counsellor. According to the MCP’s, not all women were motivated to receive smoking cessation counselling. Based on a previous study, it seems that especially women who are motivated to quit smoking and have confidence in stopping with professional support make use of support (Naughton *et al*. 2020). This underlines the importance of motivational interviewing in the smoking cessation support of pregnant women. Another possible explanation for the low referral rate can be given based on the results of our survey, which showed that women also made use of other support options, such as referral to the trained nurse practitioner or nicotine replacement therapy. These are both effective support options, as they increase smoking cessation rates (Chamberlain *et al*. 2017; Bar-Zeev *et al*. 2018).

A result of this study is that 25% of women who received counselling from addiction care stopped smoking. This effectiveness is low compared to the percentage from the Cochrane systematic review by Chamberlain *et al*. (2017) in which they report that counselling increases the likelihood of abstinence in late pregnancy with an average of 44% compared to usual care or no intervention. However, it is hard to make an exact comparison with the result of our study because we have no data about the percentage of women who stop smoking without an intervention. It is known from other literature that combination of interventions, such as counselling and nicotine replacement therapy, seems to be the most effective (Ioakeimidis *et al*. 2019).

The percentage of women who were initially referred but who ultimately did not respond to the contact attempts of the counsellors or stopped the trajectory prematurely is 20.5%. From the interviews of this study, one possible explanation emerged, namely women’s experienced pressure to be referred. The professionals might have insisted on referral so much that women agreed to be referred, even when they were not motivated to stop smoking. In the interviews, the counsellors also expressed the importance of the first meeting being face-to-face, to establish a relationship with women. This was also found in a study among oncology healthcare practitioners, which concludes that healthcare practitioners often prioritize building a strong therapeutic relationship with patients, which can lead to delaying or softening conversations about smoking cessation (Frazer *et al*. 2024). The importance of a trusting relationship for women’s adherence to support is described in other studies focusing on the patient-provider bond (Leslie and Lonneman 2016; Jordan 2020).

### Limitations and strengths

A first limitation is that 15 primary midwifery care practices (30%) and three obstetrics departments (60%) did not provide any data due to the heavy burden of the Covid-19 pandemic on their work. Therefore, the exact prevalence of pregnant women who smoke in the MCU’s is unknown. Furthermore, it is unknown how many women declined to be referred to a counsellor of addiction care. Selection bias may have influenced the results of our study, as women who were more motivated were perhaps more likely to seek counselling. Moreover, the women who identified for participation in the study were self-reported smokers. A self-reported smoking status likely underestimates the actual prevalence of women who smoke during pregnancy. This needs to be taken into consideration while interpreting the results (Ford *et al*. 1997). Furthermore, for the practices and obstetric departments that did provide data, it could be that some women are counted twice because women may be referred from primary midwifery care to secondary care in the hospital. Another limitation of this study is that we have no data about the maintenance of the implementation, the effect of coaching on women’s smoking behaviour postpartum, and women’s use of other smoking cessation support options.

A strength of the implementation is the participatory action design, with the involvement of midwives, obstetricians, experts by experience in poverty and social exclusion and pregnant women. This design increases the chance for sustainable implementation (Jagosh *et al*. 2012). Another strength is the use of mixed methods to evaluate the implementation plans, which made it possible to understand the results of the implementation in the specific context (Albright *et al*. 2013).

### Recommendations

The results indicate that despite the use of tailored implementation strategies and intermediate adaptations of the strategies, the adoption and effectiveness of the intervention can be improved. To realize this, frequent repetition of implementation strategies is necessary. Furthermore, there could be opportunities in the education of MCP’s to enhance their skills and knowledge in motivational interviewing. This could increase the rate of women who discuss their smoking behaviour with MCP’s, address the challenge of referring women who are not motivated and handles the pressure that women experience from their midwife and/or obstetrician in making use of support. More research is needed on the factors that influence referral by MCP’s, on how to improve the likelihood of women answering phone calls after a referral and how to increase the number of women that participate in the intervention. To improve the effectiveness of the counselling by addiction care, the counsellors could consider combining their counselling with nicotine replacement therapy under supervision of a doctor, feedback (e.g. by using Carbon Monoxide monitoring) or incentives (Leung and Davies 2015; Chamberlain *et al*. 2017; Ioakeimidis *et al*. 2019; Notley *et al*. 2025).

## CONCLUSIONS

In this study we developed and evaluated plans for the implementation of the guideline regarding the smoking cessation support of pregnant women in the north of the Netherlands. PAR was used as the research design to ensure that the implementation was targeted to the needs of both women and MCP’s. Seven MCU’s implemented the intervention to refer pregnant women to a counsellor from addiction care for smoking cessation support. Only a minority of the MCP’s actually referred women to a counsellor from addiction care and a small percentage of women managed to stop smoking. Opportunities in the repetition of implementation strategies and increasing skills in motivational interviewing could improve adoption of interventions in future implementation. To increase the effectiveness of the intervention, the counsellors could consider combining their counselling with nicotine replacement therapy, feedback (by using Carbon Monoxide monitoring) or incentives.

## Supplementary Material

daaf084_Supplementary_Data

## Data Availability

The datasets used and/or analysed during the current study are available from the corresponding author on reasonable request.
